# Effects of Loading Frequency and Specimen Geometry on High Cycle and Very High Cycle Fatigue Life of a High Strength Titanium Alloy

**DOI:** 10.3390/ma11091628

**Published:** 2018-09-06

**Authors:** Yanqing Li, Qingyuan Song, Shichao Feng, Chengqi Sun

**Affiliations:** 1 State Key Laboratory of Deep–Sea Manned Vehicles (China Ship Scientific Research Center), Wuxi 214082, China; lyq2006702@163.com (Y.L.); fsc0372@163.com (S.F.); 2 State Key Laboratory of Nonlinear Mechanics, Institute of Mechanics, Chinese Academy of Sciences, Beijing 100190, China; songqingyuan@imech.ac.cn; 3 School of Engineering Sciences, University of Chinese Academy of Sciences, Beijing 100049, China

**Keywords:** high cycle fatigue, very high cycle fatigue, frequency effect, specimen geometry effect, high strength titanium alloy

## Abstract

Titanium alloys have been widely used in the structural parts of deep-sea equipment and aviation industries. In this paper, the effects of loading frequency and specimen geometry on the high cycle and very high cycle fatigue life of the high strength titanium alloy Ti-6Al-2Sn-2Zr-3Mo-X is investigated by conventional fatigue test and ultrasonic frequency fatigue test. The results indicate that ultrasonic frequency could enhance the fatigue life of the highstrength titanium alloy compared with that under conventional frequency, and the frequency effect is related to the stress amplitude. This phenomenon is explained by the heat generation in specimens and heat dissipation, in combination with the high strain rate leading to the higher yield strength in the ultrasonic fatigue test. Moreover, it is indicated that the effect of specimen geometry on the fatigue life of the highstrength titanium alloy could be evaluated from the view of control volume.

## 1. Introduction

Ultrasonic fatigue testing systems have been widely used in fatigue tests, especially for very high cycle fatigue (VHCF) due to its high efficiency [1,2,3,4,5,6,7]. However, one important issue for the ultrasonic fatigue test technique is that the variation of strain rate and the heat generation in specimens produced by high cyclic loading may have great influence on the fatigue behavior of materials and lead to incorrect results. Many studies have been carried out to investigate the frequency effect by ultrasonic fatigue test. For example, Morrissey and Nicholas [8] showed that the effect of frequency including the temperature rise during ultrasonic fatigue test was negligible for a Ti-6Al-4V alloy in a VHCF regime. Takeuchi et al. [9] investigated the frequency effect on VHCF property of smooth and notched specimens of a Ti-6Al-4V alloy at different heat treatments. It was shown that the frequency effect was negligible for the specimens of Heat A and B presenting interior failure. However, as the specimens of Heat C developed only surface failure, the ultrasonic fatigue test showed higher fatigue strength than that under a conventional fatigue test. The notched specimens showed almost no frequency effect. Guennec et al. [10] showed that the ultrasonic frequency test had great influence on the fatigue strength of a low carbon steel compared to that under conventional frequency test. In their results, the frequency effect was discussed by the micro-plasticity behavior such as the stress-strain hysteresis loop and the local misorientation. The results by Morrissey et al. [11] indicated that the effect of frequency on high cycle fatigue of a Ti-6Al-4V alloy was related to the stress ratio *R*. At low stress ratio *R*, the fatigue strength increased with the increase of frequency. 

On the other hand, the fatigue strength or fatigue life of materials usually decreases with the increase of specimen size due to the fact that the larger specimens contain a higher possibility of defects and microstructure inhomogeneities [12,13]. Shirani and Härkegård [14] used Weibull’s weakest-link method to study the specimen size effect in ductile cast iron for wind turbine components. Furuya [15] investigated the effect of specimen size on VHCF behavior of high strength steels by ultrasonic fatigue test. It was indicated that the fatigue strength was lower for the specimen with larger control volume due to the appearance of larger inclusions. Sun et al. [16] assumed that a large specimen was seen as a number of small specimens via control volume and developed a method to evaluate the effect of specimen size on the fatigue life from the point of statistical analysis. It was shown that, if the fatigue life of small specimens followed Weibull distribution, the fatigue life of large specimens also followed Weibull distribution. Wang et al. [17] combined the critical distance and the highly stressed volume method to investigate the specimen size effect on the low cycle fatigue life of a TA19 titanium alloy plate. It was shown that the combining method was better than the theory of critical distance method alone.

Titanium alloys have excellent performance such as high fatigue resistance, high corrosion resistance, and high temperature resistance, and have been widely used in deep-sea equipment and aviation industries. Many studies have been performed for the microstructural, mechanical, and fatigue properties of titanium alloys [18,19,20]. Ti-6Al-2Sn-2Zr-3Mo-X is a new type of titanium alloy with high yield strength and high fracture toughness, which could be used in structural components in deep-sea equipment such as submersibles. In this work, the conventional fatigue test and ultrasonic fatigue test were first performed for this high strength titanium alloy. Then, the effects of ultrasonic frequency and specimen geometry on the high cycle fatigue and VHCF life of the titanium alloy were investigated. The effects of frequency and specimen geometry were also analyzed from the view of statistical analysis and the control volume.

## 2. Materials and Methods

**Figure 1:**
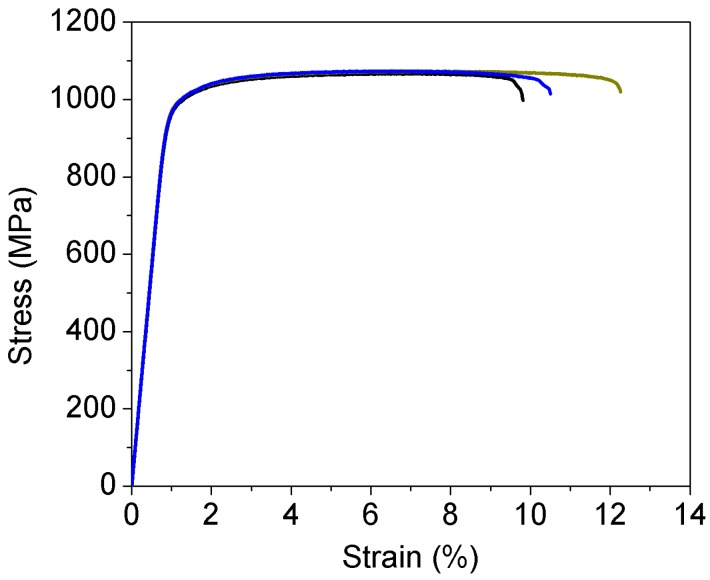
Relation of stress and strain under tensile test, in which the colors of the line denote different specimens.

**Figure 2:**
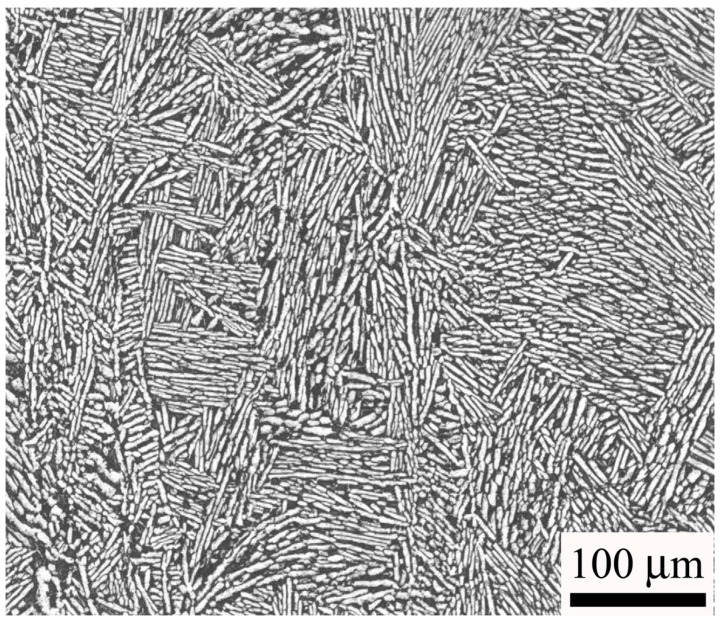
Microstructure of the Ti-6Al-2Sn-2Zr-3Mo-X alloy.

The material used was a highstrength titanium alloy, Ti-6Al-2Sn-2Zr-3Mo-X, directly cut from a forged flat plate parallel to the rolling direction. The tensile test was conducted on three cylindrical specimens with diameter of 5 mm and straight length of 30 mm in test section by the Landmark servohydraulic test system (MTS Systems Corporation, Eden Prairie, MN, USA). The relation between the stress and strain is shown in Figure 1. The tensile strength and yield strength are 1072 MPa and 978 MPa, respectively. The microstructure of the material was basket-weave consisting of α phase lamella and *β*_trans_, as shown in Figure 2. 

**Figure 3:**
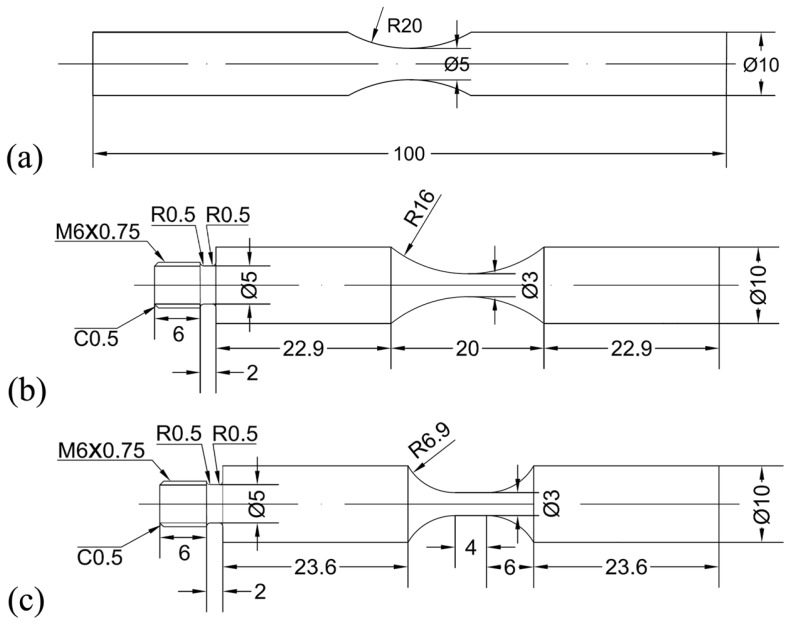
Shape and dimensions (in mm) of specimens for fatigue test. (**a**) Hourglass specimen for conventional fatigue test (HC specimen); (**b**) Hourglass specimen for ultrasonic fatigue test (HU specimen); (**c**) Dogbone specimen for ultrasonic fatigue test (DU specimen).

**Figure 4:**
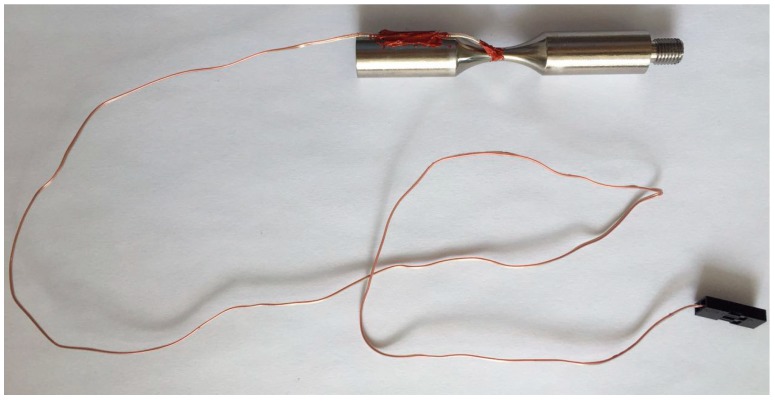
Picture of the thermocouple adhered to the surface of the small section of a HU specimen.

Three kinds of specimens with different geometry shapes were used for fatigue test, which were named as HC specimen, HU specimen and DU specimen and shown in Figure 3. The HC specimen was conducted by the MTS Landmark servohydraulic test system at room temperature. The frequency was 35 Hz and the stress ratio *R* was −1. The HU specimen and DU specimen were performed by an ultrasonic fatigue test systemUSF-2000 (Shimadzu, Kyoto, Japan) without intermittence at room temperature in air. The frequency was 20 kHz and the stress ratio *R* was −1. For the ultrasonic fatigue test, compressive cold air was used to reduce the temperature raise of the specimens during the fatigue test. A thermocouple (Yuyao Metal Electric Meter Co., Ltd., Yuyao, China) was applied for measuring the surface temperature of the small section of a few specimens, and the high-temperature adhesive YK-607 (Yikun Electronic Technology Co., Ltd., Macheng, China) was used to adhere the thermocouple and the surface of the small section of specimens, as shown in Figure 4. Before fatigue test, the surface of the test section was ground and polished in order to eliminate machine scratches. The fracture surfaces of all failed specimens were observed by the JSM-IT300 scanning electron microscope (SEM) (JEOL, Tokyo, Japan).

## 3. Results and Discussion

### 3.1. Experimental Results and Analysis

**Figure 5:**
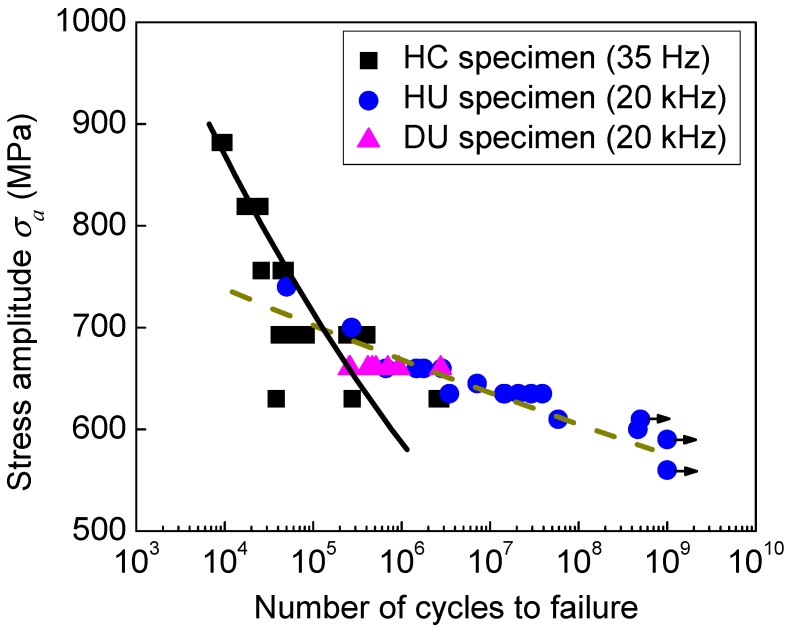
S-N data of specimens with different geometry shapes, in which the arrow denotes the specimen unbroken.

Figure 5 shows the stress-life (S-N) data of the specimens with different geometries, in which the stress concentration factor 1.05 was incorporated in the stress amplitude for the HC specimen; the solid line denotes the median S-N curve obtained from the data from the HC specimen and the dashed line denotes the median S-N curve obtained from the data of both the HU specimen and DU specimen. The original experimental data are listed in Table 1. It is seen from Figure 5 that the effect of ultrasonic frequency on the fatigue life was related to the stress amplitude. For relative lower stress amplitude, the fatigue life under ultrasonic fatigue test was longer than that under the conventional fatigue test.

**Figure 6:**
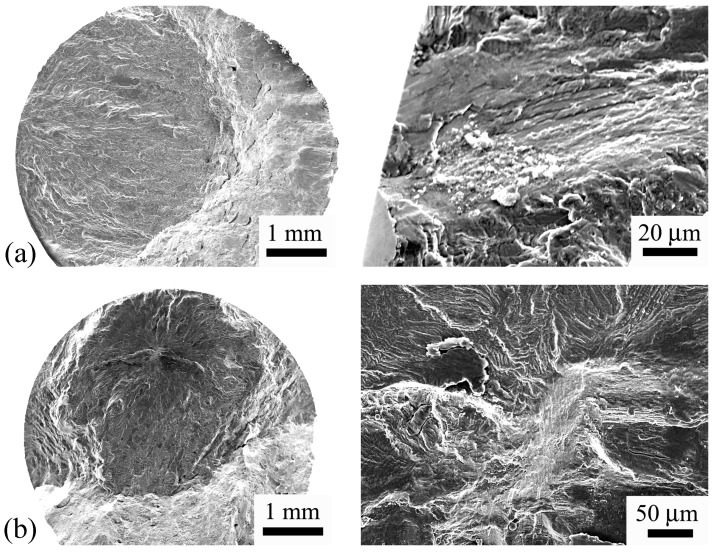
SEM observations of fracture surfaces for HC specimens. (**a**) Crack initiates from specimen surface, *σ_a_* = 693 MPa, *N_f_* = 3.27 × 10^5^; (**b**) Crack initiates from the interior of specimen, *σ_a_* = 630 MPa, *N_f_* = 2.52 × 10^6^.

**Figure 7:**
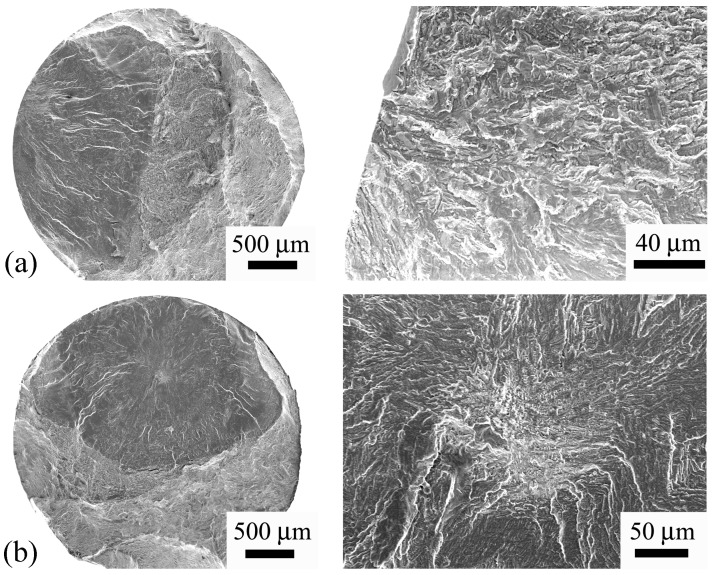
SEM observations of fracture surfaces for HU specimens. (**a**) Crack initiates from specimen surface, *σ_a_* = 635 MPa, *N_f_* = 2.91 × 10^7^; (**b**) Crack initiates from the interior of specimen, *σ_a_* = 600 MPa, *N_f_* = 4.65 × 10^8^.

Figure 6 and Figure 7 show the morphology of the fracture surface of several failed specimens under conventional fatigue test and ultrasonic fatigue test, respectively. It is seen that, for both conventional and ultrasonic fatigue tests, the fatigue failure initiates not only from the specimen surface but also from the interior of the specimen. This indicates that the crack initiation does not always occur in the maximum stress region for the present high strength titanium, which could occur at the location where the stress is lower than the maximum stress. The SEM observations of the fracture surface also indicate that the ultrasonic frequency fatigue test did not change the crack initiation mechanism compared with the conventional frequency fatigue test. It is noted that some of the fracture surfaces of the specimens under ultrasonic fatigue test burned out due to the final abrupt raise of temperature before fracture. For these specimens, the crack initiation sites were not observed.

### 3.2. Effect of Specimen Geometry

**Figure 8:**
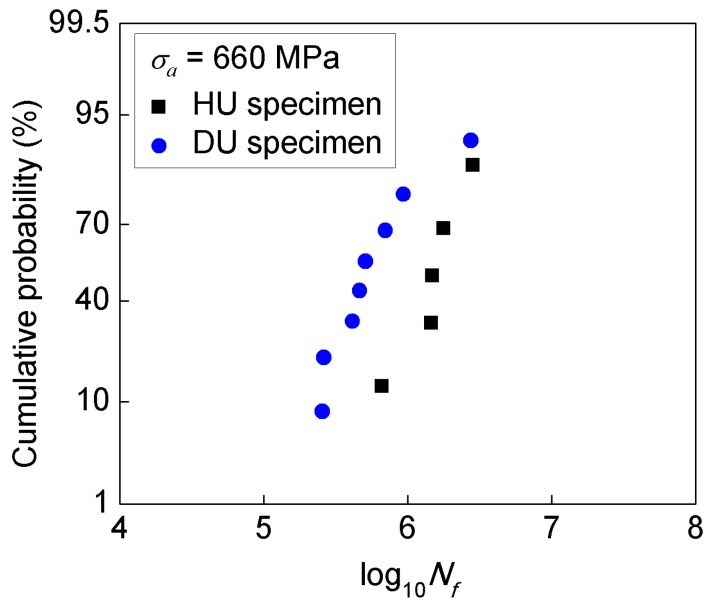
Cumulative probability of fatigue life in logarithmic scale for specimens under ultrasonic fatigue test.

For clarifying the effect of specimen geometry on fatigue life, the cumulative probability of fatigue life in logarithmic scale under the stress amplitude *σ_a_* = 660 MPa is plotted in Figure 8. The values of $\log_{10}N_{f}$ are ranked as $\log_{10}N_{f,1}\leq \log_{10}N_{f,2}\leq \ldots \leq \log_{10}N_{f,n}$, and the cumulative probability of $\log_{10}N_{f}$ no larger than $\log_{10}N_{f,i}$ is calculated by Reference [21].

(1) $$F(\log_{10}N_{f})=\frac{i-0.3}{n+0.4}$$

where *n* is the number of specimens, and *i* = 1, 2, …, *n* is the sequence number.

As shown in Figure 8, the logarithmic fatigue life scatters in the range of 5.82~6.45 for HU specimens and in the range of 5.41~6.44 for DU specimens. This indicates that the fatigue life of HU specimens and DU specimens is approximately in the same scatter band, i.e. the difference of the fatigue life is negligible when the scatter of the fatigue life is taken into account.

Further, the effect of specimen geometry on the fatigue life was analyzed in view of the control volume, which has been effectively used to correlate the specimen size effect on the fatigue strength or fatigue life [16,22,23]. Considering that the fatigue failure for the present high strength titanium initiates not only from the specimen surface but also from the interior of the specimen, the control volume was taken as the region subjected to larger than 90% of the maximum stress as used in the literature [16,22,23], which is 67.1 mm^3^ for HC specimen, 28.3 mm^3^ for HU specimen, and 34.2 mm^3^ for DU specimen calculated by finite element analysis. It is seen that the difference of the control volume for the HU and DU specimen is very small. Hence, the effect of specimen geometry on the fatigue life is negligible from the view of control volume, which is consistent with the results in Figure 8. This indicates that the effect of specimen geometry could be correlated through the control volume of the specimens for the present titanium alloy. 

### 3.3. Ultrasonic Frequency Effect on Fatigue Life

According to the results in Section 3.2, the effect of specimen geometry on the fatigue life of the present titanium alloy could be correlated through the control volume of specimens. For the present three types of specimens with small differences of control volume (the control volume of HC specimen is 2.37 times that of the HU specimen, and is 1.96 times that of the DU specimen), the effect of specimen geometry is not taken into account when dealing with the effect of frequency on the fatigue life. Considering the scatter of fatigue life, the median S-N curves under both the conventional fatigue test and the ultrasonic fatigue test were obtained using the method by Sun et al. [16] and were used for analyzing the effect of frequency, as shown in Figure 5. It is seen that the effect of ultrasonic frequency on the fatigue life is related to the stress amplitude. For some stress levels, the frequency effect might be negligible. While for the relative lower stress level, the ultrasonic frequency improves the fatigue life, and this effect tends to increase with the decrease of the stress amplitude. 

**Figure 9:**
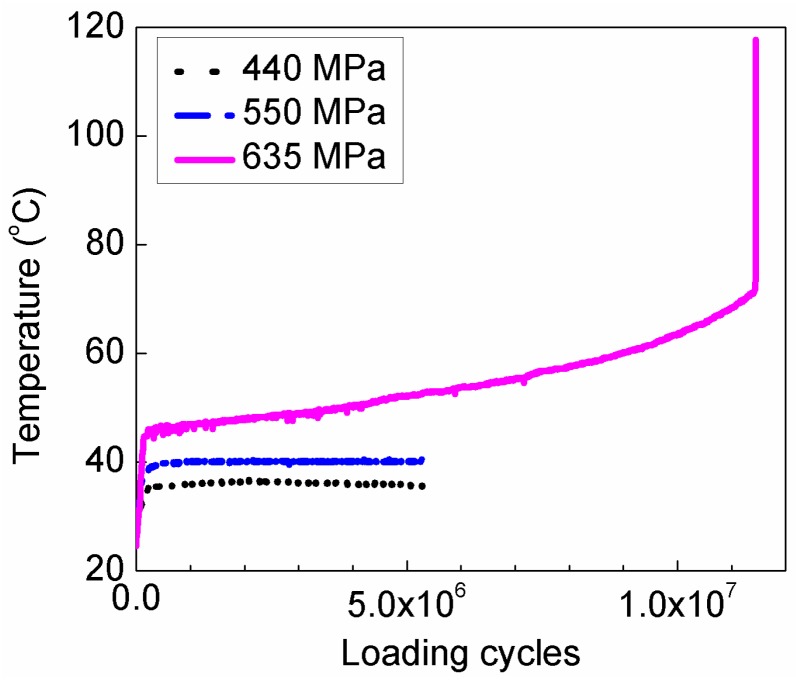
Variation of the surface temperature of the smallest section of HU-specimens with loading cycles during ultrasonic fatigue test.

As is well known, one important factor influencing the validity of ultrasonic fatigue tests is that the internal heat generation in specimens during the fatigue test might lead to inaccurate results in high cycle and VHCF regimes. Figure 9 shows the variation of the surface temperature of the smallest section versus the loading cycles for HU specimens during ultrasonic fatigue test. It is seen from Figure 9 that the surface temperature of the small section of specimens strongly depends on the stress amplitude, which increases with increasing the stress amplitude. For relative lower stress amplitude (400 MPa and 550 MPa), the temperature stabilizes very rapidly (at the loading cycles lower than 5 × 10^5^), and the stable temperature is just a little higher than the room temperature (25 °C). So, the effect of self-heating in specimens might be negligible at the lower stress amplitude (e.g., less than 550 MPa). While for relative higher stress amplitude, the temperature increases sharply at the first hundreds of thousands of loading cycles, then increases slowly with the loading cycles, and does not stabilize even up to 10^7^ cycles. This indicates that the heat dissipation is effective for the lower stress amplitude by the compressive cold air cooling system and the temperature of specimens are cooled down. However, for the higher stress amplitude, the generated heat due to the local plastic deformation and internal friction cannot be dissipated effectively under the identical cooling system, which induces the decrease of fatigue life [24]. On the other hand, the high strain rate in the ultrasonic fatigue test leads to the higher lower yield strength and enhances the fatigue life [25,26]. These factors together result in the difference of the S-N curves under conventional frequency and ultrasonic frequency fatigue test, as shown in Figure 5.

## 4. Conclusions

This paper studies the effects of loading frequency and specimen geometry on the high cycle fatigue and VHCF life of the high strength titanium alloy Ti-6Al-2Sn-2Zr-3Mo-X by conventional fatigue test and ultrasonic frequency fatigue test. It was shown that the ultrasonic frequency effect on the fatigue life is related to the stress amplitude, although it does not change the crack initiation mechanism compared with the conventional frequency fatigue test. The difference of the S-N curves under the conventional frequency fatigue test and ultrasonic frequency fatigue test is explained by the heat generation in specimens and heat dissipation, in combination with the high strain rate in ultrasonic fatigue test which leads to the higher yield strength. 

Moreover, the paper indicates that the control volume method could be used for correlating the effect of specimen geometry on the fatigue life of the high strength titanium alloy.

## Figures and Tables

**Table 1:** Original experimental data from the specimens shown in Figure 5.

HC Specimen		HU Specimen		DU Specimen	
*σ_a_* (MPa)	*N_f_*	*σ_a_* (MPa)	*N_f_*	*σ_a_* (MPa)	*N_f_*
882	8.98 × 10^3^	740	4.99 × 10^4^	660	2.75 × 10^6^
882	9.40 × 10^3^	700	2.72 × 10^5^	660	9.34 × 10^5^
882	9.82 × 10^3^	660	2.84 × 10^6^	660	4.64 × 10^5^
819	1.79 × 10^4^	660	1.78 × 10^6^	660	4.13 × 10^5^
819	2.48 × 10^4^	660	1.48 × 10^6^	660	2.61 × 10^5^
819	1.82 × 10^4^	660	1.46 × 10^6^	660	6.99 × 10^5^
819	1.70 × 10^4^	660	6.64 × 10^5^	660	2.55 × 10^5^
756	4.34 × 10^4^	645	7.10 × 10^6^	660	5.10 × 10^5^
756	4.36 × 10^4^	635	3.88 × 10^7^	-	-
756	2.58 × 10^4^	635	2.91 × 10^7^	-	-
756	4.83 × 10^4^	635	4.09 × 10^7^	-	-
693	2.39 × 10^5^	635	1.46 × 10^7^	-	-
693	6.45 × 10^4^	635	1.42 × 10^7^	-	-
693	3.27 × 10^5^	635	3.46 × 10^6^	-	-
693	7.97 × 10^4^	610	5.85 × 10^7^	-	-
693	4.04 × 10^5^	600	4.65 × 10^8^	-	-
693	4.16 × 10^4^	610 *	5.0 × 10^8^	-	-
693	4.90 × 10^4^	590 *	1.0 × 10^9^	-	-
693	8.29 × 10^4^	560 *	1.0 × 10^9^	-	-
630	2.74 × 10^5^	-	-	-	-
630	2.77 × 10^6^	-	-	-	-
630	2.52 × 10^6^	-	-	-	-
630	3.82 × 10^4^	-	-	-	-

* It denotes the specimen remained unbroken.
